# Positive Mental Health and Mental Health Literacy of Informal Caregivers: A Scoping Review

**DOI:** 10.3390/ijerph192215276

**Published:** 2022-11-18

**Authors:** Carmen Andrade, Márcio Tavares, Hélia Soares, Fábio Coelho, Catarina Tomás

**Affiliations:** 1 Department of Nursing, Family and Community Health, School of Health, University of the Azores, 9500-321 Ponta Delgada, Portugal; 2 Group Inovation and Development in Nursing (NursID), Centro de Investigação em Tecnologias e Serviços de Saúde (CINTESIS), 4200-450 Porto, Portugal; 3 Department of Nursing, Mental Health and Gerontology, University of the Azores, 9700-042 Angra do Heroísmo, Portugal; 4 Nursing Research Unit for South and Islands (NURSE’IN), 2914-503 Setubal, Portugal; 5 Department of Nursing Sciences, School of Health, Polytechnic Institute of Leiria, 2411-901 Leiria, Portugal; 6 ciTechCare-Center for Innovative Care and Health Technology, School of Health Sciences (ESSLei), Polytechnic of Leiria, 2411-901 Leiria, Portugal

**Keywords:** positive mental health, mental health literacy, caregivers, review literature

## Abstract

Positive mental health and mental health literacy are two main concepts to ensure an individual and social state of mental health and well-being. A scoping review of the scientific literature published in the field of health sciences was conducted to identify the relationship between mental health literacy and the positive mental health of family caregivers. A research expression was used to search for articles in health databases, respecting the main topics of the Participants/Concept/Context (PCC) framework. A total of eight articles were included from the 2830 initially identified using the PRISMA Extension for Scoping Reviews (PRISMA-ScR) process. It was noticeable that none of the studies related positive mental health and mental health literacy of caregivers. Nevertheless, it was possible to identify predictors of mental health and self-efficacy, such as burden and a lack of information about and support in the process of care. Caregivers’ quality of life, self-esteem and confidence are also important positive mental health predictors that are closely related to health literacy. The knowledge of these factors can contribute to the reduction in negative determinants of mental health of caregivers and the resolution of strategies to meet caregivers’ needs.

## 1. Introduction

Mental illness is considered one of the main causes of morbidity worldwide, and problems associated with mental health account for 12% of the global burden of disease. Every year, 165 million people in Europe are diagnosed with a mental illness or disorder [1].

Mental health is decisive for personal, social and socioeconomic development. Despite the scientific evidence that accounts for this reality, the failure to achieve the goals set in the 2013–2020 action plan of the World Health Organization (WHO) for the area of mental health leads to the conclusion that the attention and investment given to the promotion of mental health are still insufficient in most countries [2]. In this regard, the United Nations, in the Sustainable Development Goals for 2030, refers to the promotion of mental health and well-being as one of the necessary goals of intervention, crucial to transform the world in a more sustainable context, particularly in the third goal, which aims to ensure access to quality health services and to promote well-being for all at all ages [3].

Mental health is an important part of health and is one of the pillars that supports especially individual well-being but also the health of the society in which people live. The state of mental well-being allows people to be able to cope with life challenges, make decisions, build relationships, learn, develop their skills, and become citizens with an active role in the community where they live [4,5]

Positive mental health (PMH) is associated with the promotion of personal skills and involves a dynamic state of positive and negative emotions, thoughts, and behaviors that promotes individual qualities [6]. That means that through the acceptance of these emotions, the person can become resilient by maximizing an optimistic and problem-solving perspective. In this regard, Lluch [6] developed a multifactorial model of PMH where essential factors/characteristics are highlighted for maintaining and ensuring a positive state of mental health and for coping with and resolving problems in daily life.

This same author states that this model of PMH includes six factors: (1) Personal Satisfaction (self-concept/self-esteem, satisfaction with one’s own life); (2) Pro-Social Attitude (helping-supporting attitude towards others, acceptance of others and distinct social facts); (3) Self-Control (ability to cope with conflicting situations, emotional balance/emotional control, tolerance of frustration, anxiety, and stress); (4) Autonomy (independence, self-regulation of one’s own behavior, ability to have one’s own criteria, personal security/self-confidence); (5) Problem Solving or Self-Realization (analytical skills, ability to make decisions, flexibility/ability to adapt to changes, attitude of continuous personal growth and development); (6) Interpersonal Relationship Skills (ability to establish interpersonal relationships, ability to provide emotional support, empathy/ability to understand others’ feelings, ability to establish and maintain close interpersonal relationships).

Despite the difficulty in finding an exact definition of PMH, it can be understood as the ability for the individual to perceive him/herself and to recognize the environment and the community as facilitating factors, and thus engage and adapt to it in an optimistic way [7]. Therefore, PMH is considered a means through which individuals are protected from the development of mental disorders, allowing them to have a full life with him/herself and with society [8].

Although health policies and health programs that contribute to the support of the informal caregiver are being increased in some countries, being a family caregiver of a person with mental illness may pose a risk to the caregiver’s own health due to the challenges at different levels (e.g., personal, relational, financial, and organizational). Thus, in addition to health policy measures, it is essential to develop intervention programs focused on the needs of the informal caregiver, specially aimed at promoting their health and well-being and increasing mental health literacy [9].

Mental health literacy (MHL) was initially defined as the “knowledge and beliefs about mental disorder, its recognition, management and prevention” [10] (p. 182). Later, the author describes this knowledge more broadly by involving the ability to recognize and distinguish different mental disorders, their risk factors, and causes of those disorders; knowledge of accessible forms of self-help and professional help; attitudes that can promote appropriate help; and knowledge of ways to obtain relevant health information [11]. Thus, mental health literacy has become a prerequisite for recognition and early intervention in mental disorders [12].

The available knowledge on the issue of family caregivers is consensual in recognizing that caring for a dependent person implies great exhaustion, with consequences on their health [13,14,15]. The exhaustion of having to deal with various tasks and responsibilities can lead to states of anxiety, depression, panic, and loneliness that ultimately have an impact on physical, mental, emotional, social, and economic conditions [16]. The degree of burnout and the impact on the caregiver’s health depend on their coping and adaptation strategies to the situation; in particular, more information on how to care for the person with physical and mental dependence is related to lower levels of overload [15].

The knowledge and use of those strategies depend on the MHL level: the higher the level, the greater the knowledge and ability to respond appropriately to the challenges and difficulties related to the situation of caring for someone who is dependent [17]. People with higher levels of positive mental health literacy (MHL+) are more predisposed to engage in self-care and search for better resources with their family, the social support structure, the community, and the health system [18]. Therefore, we believe that the intervention of health professionals from a salutogenic and socio-ecological perspective with family caregivers, considering the individual, social, and contextual factors that influence the MHL+, will determine a greater motivation and competence to access, understand, assess, and apply information. This increase in MHL will have effects on the increase in PMH regarding problem-solving competence, autonomy, personal satisfaction, interpersonal relationships, self-control, and pro-social attitude. To this extent, there is an urgent need for the development of programs within PMH that aim at empowering individuals to promote positive feelings and self-control skills.

Taking this into account, this scoping review aimed to review the scientific literature published in the field of health sciences to identify the relationship between the positive mental health of family caregivers and their MHL. We expected to identify how the levels of PMH can be affected by the MHL so as to evolve research and interventions according to the conclusions.

## 2. Materials and Methods

This scoping review was conducted in March 2022. This methodology was chosen due to its exploratory nature and because it allows researchers to identify and synthetize evidence on a specific topic that has been explored in a limited way, which seems to be the case for this study [19]. Thus, to answer the initial question outlined, “What is the relationship between mental health literacy and positive mental health of family caregivers of dependent persons in home settings?”, articles available in scientific databases were selected, as they are considered reliable analytical tools that ensure quick access to relevant peer-reviewed papers. The recommendations of the Joanna Briggs Institute Reviewer’s Manual were followed, following the instructions of Tricco et al. [20] in the use of the PRISMA Extension for Scoping Reviews (PRISMA-ScR).

Following this main aim, the goals of this review were to identify the levels of PMH in family caregivers and the determinants that interfere with those levels, to identify informal caregivers’ levels of MHL, and to relate the levels of MHL and PMH in these caregivers.

To define the research expression, we used the PCC framework, which determines that the population (P) is informal caregivers of dependent persons, the concept (C) is the PMH and its relationship with mental health literacy, and the context (C) is the home care context.

After the definition of search terms, extracted from the descriptors of Medical Subject Headings (MESH) and Descritores em Ciências da Saúde (DECS), terms were conjugated with a logical expression, using the Boolean operators “AND” and “OR” and additional instruments such as “()” and “*”. The final research expression used was [(“Mental Health” OR “Mental Hygiene” OR “Positive Mental Health”) AND (Caregiver* OR “Famil* caregiver*” OR “Informal caregiver*” OR Carer* OR “Spouse caregiver*”) AND (“Mental Health Literacy” OR “Health Literacy”)].

The research was carried out using several health databases, namely Academic Search Complete, CINAHL Plus with Full Text, MEDLINE with Full Text, MedicLatina, eBook University Press Collection (EBSCOhost), eBook Collection (EBSCOhost), Psychology and Behavioral Sciences Collection, and PubMed.

The articles to include in the research were selected using inclusion and exclusion criteria. We included articles that included the assessment of the informal caregivers’ MHL or PMH, articles that related MHL and the family caregivers’ PMH, and articles with peer review. From those, we excluded articles published before 2017; articles not written in English, Portuguese, or Spanish; literature reviews or opinion articles; and articles whose study focus was informal caregivers of children under 18 years of age.

The article selection process followed the PRISMA flowchart. It should be noted that during the process of study analysis, two independent reviewers performed the critical appraisal, extraction, and synthesis of data, and in case of disagreement, a third reviewer was consulted for analysis and to make the decision of inclusion or exclusion.

## 3. Results

**Figure 1:**
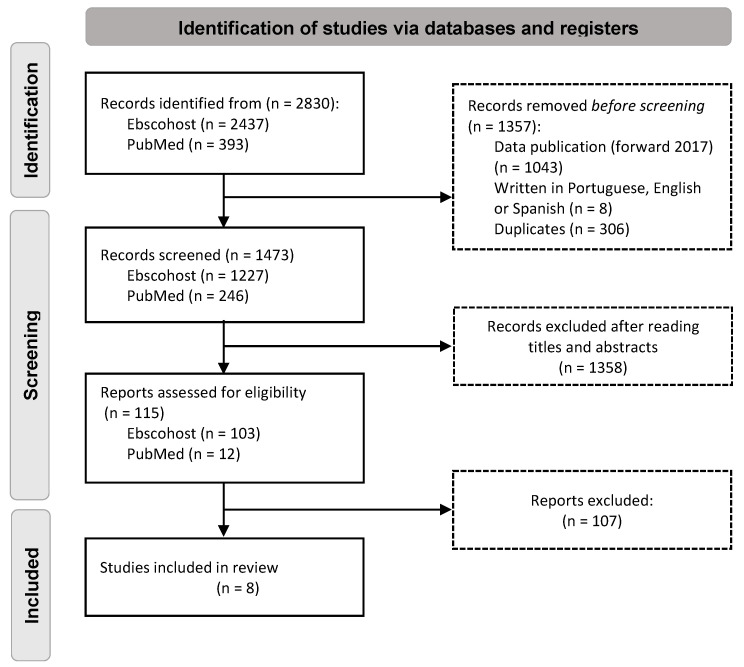
Study flow diagram according to the PRISMA.

From the database search, 2830 articles were initially identified between the search years 2017 and 2022. From this total, 1357 articles were excluded, namely for being published prior to 2017 (*n* = 1043), for not being written in Portuguese, English, or Spanish (*n* = 8), or for being duplicate articles (*n* = 306). The remaining articles, a total of 1473, were screened based on title and abstract, resulting in the exclusion of 1358 articles. The remaining 115 articles were subjected to full-text reading, resulting in the exclusion of 107 using the exclusion and inclusion criteria. Thus, eight articles published between 2018 and 2022 met the defined inclusion criteria and were included in the review process. Figure 1 shows a schematic representation of the article selection process according to the PRISMA guide for reporting systematic reviews. Since the different studies stemming from the included articles varied in methodology, participants, and outcome measures, it was considered not to undergo meta-analysis. 

The evaluation of the quality of the articles was performed according to the GRADE (Grading of Recommendations Assessment, Development and Evaluation) assumptions [21], developed to classify the quality of evidence and the strength of recommendations in health, which represents the confidence in the information used.

According to the GRADE, of the eight studies evaluated, one has a low level of evidence (#7), five have a moderate level (#1, #2, #3, #4, and #8), and two have a high level (#5 and #6), and for that, all were included.

### 3.1. Characterization of the Studies

Of the eight studies included, two are from the United States of America, two are from China, and the remaining four are from Germany, France, India, Indonesia, and Nigeria. A total of 1329 family caregivers were included in the samples of the eight studies. Only one study did not include sociodemographic data. Considering the seven studies that refer to gender, 64.5% of the family caregivers are female; the average age based on the four studies that present this information is 49.78. Regarding the caregiver’s relationship to the family member being cared for, considering the six studies that refer to this information, the caregivers were mostly spouses (35.4%) and their children (33.3%).

As for the condition of the person being cared for, three studies identified the diagnosis of mental illness, two studies identified the diagnosis of cancer (one of them in the terminal phase), and the remaining studies referred to diagnoses of dementia, brain injury, stroke, and cognitive impairment; one study did not refer to the condition of the person being cared for (Table 1).

Three studies address family caregiver health literacy, exploring its association with caregiver burden and/or quality of life [24,25,26]. Two studies focus on the experience of caring for a dependent person with mental illness in terms of the knowledge/skills needed for the role and the mental health indicators of the family caregiver [22,28]. Two studies evaluate the impact of an intervention program with family members: one study identifies the effect of a family caregiver respite program on their confidence and well-being; the other study evaluates the effects of a support program on the health literacy and psychosocial health of family caregivers [23,29]. One study focuses on the stress and burden of family caregivers of people with the diagnosis of schizophrenia [27].

As for the study design and methods used, six had a quantitative approach [22,23,24,25,26,27]. Of the remaining studies, one took a qualitative approach using interviews and thematic data analysis [28]; one study used a mixed approach, using pre- and post-test methodology [29]. Data collection using quantitative methods was carried out using a questionnaire (namely to collect socio-demographic data) and/or using scales to measure the phenomena under study [22,23,24,25,26,29] (Table 2). In terms of the quality of the studies, all of them were classified as approved.

### 3.2. Main Results: Mental Health Literacy and Positive Mental Health

In the three studies in which health literacy and the mental health of family caregivers were assessed, it is possible to realize:

1. In the study in which the association between e-health literacy and caregiver burden was examined [24], a positive association was found between the two (β = 0.14; 95% confidence interval [0.03, 0.27]).
2. In the study that aimed to identify the levels of health literacy and explore associations with the quality of life of family caregivers and their perceptions about the role [25], 78.6% of family caregivers had high levels of health literacy, and in the group of family caregivers with low literacy (21.4%), significant effects were identified in the subjective caregiving burden (*p* = 0.041), relationship satisfaction with the patient (*p* = 0.028), and caregiving mastery (*p* = 0.030), with no difference between the groups regarding quality of life and general mental health.
3. In the study examining the association between quality of life (QoL) and family caregivers’ health literacy [26], the QoL mean score was 180 (of a possible 370); the lowest QoL scores were reported for physical well-being (26.5 ± 7.9), followed by spiritual well-being (35.5 ± 10.7). Cancer health literacy was significantly associated with QoL among participants. While QoL was negatively associated with psychosocial needs (r = −0.55, *p* < 0.01), QoL was positively correlated with cancer health literacy (r = 0.19, *p* = 0.04). QoL was significantly correlated (negative) with five of the seven domains of psychosocial needs of family caregivers; the most highly correlated domain was spiritual support (r = −0.30, *p* < 0. 001), followed by psychological problems (r = −0.24, *p* < 0.01), information (r = −0.24, *p* < 0.01), and family/social support (r = −0.19, *p* = 0.04). Approximately 28% of the variance in quality of life is explained by information practices, psychological problems, and health literacy, with information needs making one of the strongest contributions (2.9%).

As for the two studies that explored the family caregiver’s experience in role performance:

1. The study that aimed to investigate the characteristics of the caregiving experience according to age at onset of dementia to adapt support programs [22] shows confidence scores in role performance, namely regarding “Request respite care”, with scores of 51.14 (EOD) and 45.93 (LOD) out of 100; “Cope with behaviors”, with scores of 73.96 (EOD) and 71.15 (LOD); and “Control disturbing thoughts”, with scores of 65.41 (EOD) and 73.28 (LOD). As for the caregiver’s level of distress, the family caregivers presented “Psychological distress”, with scores of 26.17 (EOD) and 23.9 (LOD); “Anxiety”, with scores of 8.79 (EOD) and 7. 74 (LOD); and “Depression”, with scores of 5.89 (EOD) and 5.64 (LOD). The family caregivers showed a positive impact of “caregiving experience” with scores of 27.7 (EOD) and 26.98 (LOD) at the level of caregiver’s esteem and scores of 51.47 (EOD) and 50.3 (LOD) at the level of “Role confidence”. All caregivers were confident in performing their role, reasonably well prepared for future needs, and reported mild depressive and anxious symptoms. However, they lacked informal support, had little confidence in requesting respite care, and reported effects on their health. Comparing the two groups of caregivers, those caring for people with early dementia had more severe perceptions of the cognitive disturbances of people with dementia and reported a better sense of preparation and knowledge of services. Caregivers of spouses with late dementia were more confident in their abilities to control disturbing thoughts. The results also suggest that programs should provide information about support networks to improve the preparedness of caregivers of spouses with early dementia, as well as emphasize positive coping strategies for caregivers to maintain quality relationships with the dependent relative, which influence perceptions of symptoms.
2. From the quantitative study [28] aiming to explore the experiences of family caregivers of people with schizophrenia in outpatient treatment, themes related to family caregivers’ mental health such as emotional burden and anxiety about the future emerge from the thematic analysis.

As for the two studies assessing the impact of an intervention program on family members:

1. The study aimed at measuring the impact of a respite care program on the well-being of family caregivers [23] revealed improvements at the level of well-being indicators with scores of 3.31 (BR)/2.36 (DR) in terms of stress levels, 2.56 (BR)/1.9 (DR) for general health problem status, 2.02 (BR)/2.88 (DR) in terms of social/recreational activities, 1.83 (BR)/1.46 ± 0.82 (DR) for placing the care recipient in out-of-home care, and 5.24 (BR)/3.06 ± 3.09 (DR) in terms of stress-related health symptoms.
2. The study under an intervention program aiming to understand the perceived health literacy and psychosocial health outcomes of family caregivers [29] shows an increase after intervention; in terms of health literacy, there was an increase in the scores (T0 and T1) at the level of functional health literacy (knowledge) with 3. 7(T0)/4.2 (T1), of interactive health literacy (capability to act) with 3.3 (T0)/3.7 (T1), and of critical health literacy (individual empowerment) with 3.0 (T0)/3. 2 (T1). As for psycho-social health, there is also an increase in scores namely in terms of sense of certainty with 3.0 (T0)/3.2 (T1) and life balance with 2.8 (T0)/2.9 (T1).

Study #6 focused on the stress and burden of family caregivers of people with the diagnosis of schizophrenia [27] with the aim of assessing the degree of stress and burden among caregivers of family members with schizophrenia and early psychosis. Family caregivers showed significant psychosocial stress and burden, with a mean stress score of 3.56 (out of 5) and a mean QoL score for each of the variables >3 (out of 5), with a mean score of 3.10 for personal mental health and of 3.12 for social life. At the end of the survey, participants were asked if they had adopted any strategies for coping with stress: 46.3%, 39.6%, 26.9%, and 15.4% of caregivers reported that they had sought support from religion, social workers, services in the community, and professional psychological therapy, respectively. Approximately 26.9% of participants adopted no coping strategy. Most caregivers thought that the following measures would be markedly helpful in alleviating their stress: enhancement of community support services for the mentally ill and their caregivers (57.3%), an increase in the efficiency of services provided by psychiatric hospitals and clinics (53.7%), and an increase in the efficiency of services offered by medical social workers (50.9%).

## 4. Discussion

Based on the analysis of the identified studies, none related caregivers’ PMH to MHL. However, it is possible to identify predictors that influence their mental health and that are important to acknowledge for the purpose of this review. Talking about caregivers’ positive mental health is necessary, not only because of the emerging and growing nature of the caregiving phenomenon, but also because of the lack of studies addressing this topic. Concomitantly to the reality of the aging of the world’s population comes the problem of increasing dependence because of the growing prevalence of chronic degenerative diseases and the consequent functional limitations, but also because of advances in health care [30]. This phenomenon unequivocally leads to an effective increase in the number of caregivers due to the pressure of social systems, leading families to organize themselves and take responsibility for the task of care [31].

Thinking about the phenomenon of dependence requires reflecting on who claims the care, namely the informal caregiver, defined as someone, family or not, who takes responsibility for ensuring the satisfaction of the needs presented by the person being cared for [32].

Taking responsibility for caring for a person requires the combination of two conditions: availability to provide care and adequacy to do so [33]. In fact, assuming the responsibility of caring for a person with dependencies may signify, for those who are not prepared, a largely negative impact on their well-being, characterized as a state of burden [32,33]. When the burden of care exceeds the capacity of the informal caregiver, then situations of anxiety (#7, #1), depression (#1), fear (#7), and stress (#1, #2, #6) may arise. These are indicators that mental health may be compromised. In this regard, study #7 points to emotional distress because of the responsibility of caring for people with schizophrenia. Feelings such as fear and anxiety about what the future may hold for them are pointed out. Regarding caregiver attrition, study #1 seeks to understand if the age of caregivers might be a determinant in caregiving. They concluded, however, that age is not significantly related to caregiver stress levels or the manifestation of anxiety and depression. This suggests that caregivers’ mental health engagement is unrelated to age. In the opposite direction, the variables that actually influence this dimension are a lack of information and support networks and preparation for the responsibility of caregiving, adding to the importance of emphasizing coping strategies (#1).

Regarding stress, articles #7, #1, #2, and #6 reveal that this emotional state predominates among the caregivers who participated in the studies. Stress seems to be perpetuated by several factors, such as the responsibility of caring, the permanent and long-term nature of the care, the absence of professional or other support, isolation, financial constraints (#7, #1), fatigue, sleep disturbances, tiredness (#1, #2), headaches, muscle tension or pain (#2), and psychological overload (#6). These findings are corroborated by several studies that have been conducted over the years, in which several of these factors are pointed out as responsible for this emotional state [32,34,35].

It should be noted that these aspects directly influence caregivers’ self-efficacy. This is an important construct associated with mental health, perceived as the belief that a person has about his/her abilities to perform and organize tasks with the desired effect [36]. Stress is identified as an adversity that may affect self-efficacy (#7, #1, #2, #6), making caregivers vulnerable. At the same time, the lack of knowledge about the disease (#7) and the burden (#7, #3, #6) seem to be situations that affect this competence. The lack of knowledge about what causes the dependence of the other person may constitute an obstacle to the provision of adequate care. On the other hand, it may lead to a lack of understanding of the needs that may exist—both their own and those of the person being cared for. The context of caring is complex and involves many factors associated with the fulfilment of the caregiver’s role which, if not mastered, may represent a serious public health problem due to the consequences for the caregiver and the person under his/her care [37,38,39].

Knowledge about the issues related to the disease makes the caregiver and the person receiving care achieve better results in the quality of their lives. Thus, the mastery over the health condition of the person cared for and the necessary care allows the caregiver to perform his/her role safely [38,39].

Another aspect that seems to influence the caregiver’s self-efficacy is the burden to which they are subjected (#7, #3, and #6). In fact, the emotional burden (#7), the psychosocial burden (#6), and the physical burden [32] are identified as factors inhibiting the caregiver’s well-being and, as such, limiting self-efficacy. Therefore, it is understood that by ensuring greater social and professional support to informal caregivers and clarifying their needs, it is possible to agree on strategies that aim to improve and maintain their quality of life [32,39]. These aspects necessarily intersect with literacy issues, and those with lower levels of literacy show higher levels of subjective burden, less satisfaction in the relationship with the family caregiver, less mastery in care provision, and worse physical health (#3). However, both study #4 and study #8 seem to indicate that increased literacy supports the caregiving process. They therefore suggest designing tailored interventions for informal caregivers with low health literacy, targeting both the provision of care and the receipt of care (#4 and #8).

Available studies report that more than one-third of paid non-family caregivers had low levels of literacy and performed tasks regardless of their abilities, and low literacy was associated with worse behavior management for care patients [40]. Thus, low caregiver health literacy has the potential to impact the provision of appropriate caregiving, consequently promoting negative health outcomes in care patients and caregivers [34,40,41,42].

A caregiver’s quality of life (#5), self-esteem (#1), and confidence (#1 and #2) are dimensions that appear in the studies analyzed and seem to suggest positive mental health predictors that are closely related to health literacy. The studies focus on the importance of caregiver training and information as a guarantee of these variables. As several studies report [34,41], the analyzed articles emphasize that the trust of and in the caregiver is closely connected to the demonstrated and acquired competence (#1 and #2). The greater the expertise, the greater the security demonstrated and, consequently, the higher the caregiver’s self-esteem [43]. To reduce the impact of caregiving, it is necessary to improve the caregiver’s mastery of caregiving and self-esteem. This suggests that training should be provided to family caregivers to increase their caregiving competence and provide counseling to improve their self-esteem [35,43].

Coordinated interventions designed to meet caregivers’ needs will ensure better satisfaction with caregiving practice, contributing to improved quality of life [35]. Studies highlight the need for greater support for caregivers to increase the quality of care that is provided [35,37,43].

## 5. Conclusions

This literature review made it possible to understand that there are few studies carried out within the scope of PMH, and none of the studies found a direct relationship between PMH and MHL of caregivers.

As mental health is considered the cornerstone for the well-being of individuals and the community, the stigma associated with mental illness and the lack of MHL are important issues in the mental health area, being considered major obstacles to the promotion of mental health. MHL is understood as the recognition of mental disorders and the knowledge and attitudes promoting appropriate help, and can be experienced by family caregivers.

As the population grows older, there is an increase in disabling chronic diseases and a consequent increase in caregivers to ensure that the needs of the person being cared for are met.

It was found that caregivers may experience a set of indicators that can compromise their mental health, such as emotional exhaustion, anxiety, depression, fear, and stress, which are not related to the caregiver’s age, but rather to the lack of knowledge and preparation; isolation; financial constraints; tension; psychological, emotional, and physical overload; and lack of coping mechanisms.

In this context, it seems evident that the level of literacy on disease-related aspects has a potential influence on the achievement of effective health outcomes.

The promotion of quality of life, self-esteem, confidence, and caregiver literacy appear to be indicators of positive mental health.

As limitations of this scoping review, we consider the lack of studies that characterize the PHM and MHL of family caregivers and studies that relate these two concepts. Furthermore, most of the studies included are focused on dependent people with mental illness, which gives us a restricted perspective of a specific group of caregivers. It is important to state that the indicators found in the studies are more focused on mental health commitment and not on positive mental health indicators.

It seems that there is still a long way to go in family caregiver mental health. However, the knowledge of factors with potential influence on the caregiver’s mental health, namely those that can promote an effective PMH, may contribute to the mitigation of the complicating aspects of this process, as well as to the resolution of strategies that can meet the caregivers’ needs, promoting their mental health, a better quality of life, and, consequently, ensuring better satisfaction among all parties.

## Figures and Tables

**Table 1:** Identification and characteristics of the samples used in selected studies.

Study Identification		Sample	
Authors (Year) Location [Reference] No.	Title	Caregivers	Dependent Person’s Diagnosis
Wawrziczny, E.; Berna, G.; Ducharme, F.; Kergoat, M.; Pasquier, F.; Antoine, P. (2018) France [22] #1	Characteristics of the spouse caregiving experience: comparison between early- and late-onset dementia	*n* = 150 Female (31); Mean age = 58.98	Dementia
Ackerman, L.; Sheaffe, L. (2018) USA [23] #2	Effects of respite care training on respite provider knowledge and confidence, and outcomes for family caregivers receiving respite services	*n* = 102 Spouse (21); Children of the person cared for (20)	Not specified
Wang, K.; Gao, X.; Sun, F.; Bishop, N. (2021) China [24] #3	eHealth literacy and caregiver burden among chinese caregivers of older adults with cognitive impairment: does education matter?	*n* = 448 300 Female (300); Mean age = 57.82; Spouse (208); Children (77)	Elderly with cognitive impairment
Hahn, E.; Boileau, N.; Hanks, R.; Sander, A.; Miner, J.; Carlozzi, N. (2021) USA [25] #4	Health literacy, health outcomes, and the caregiver role in traumatic brain injury	*n* = 131 Female (103); Mean age = 46.2; Spouse (62); Children (11)	Traumatic brain injury
Gabriel, I.; Creedy, D.; Coyne, E. (2020) Nigeria [26] #5	Quality of life and associated factors among adults living with cancer and their family caregivers	*n* = 120 Female (85); Mean age = 36.13; Spouses (88)	Cancer
Wan, K.; Wong, M. (2019) China [27] #6	Stress and burden faced by family caregivers of people with schizophrenia and early psychosis in Hong Kong	454 Female: 326 Age = 34.8% with more than 46 years	Schizophrenia and psychosis
Nuraini, T.; Tumanggor, R.; Hungerford, C.; Lees, D.; Cleary, M. (2021) Indonesia [28] #7	Caregiver burden for people with schizophrenia in Medan, Indonesia	*n* = 10 Female (5); Spouse (6); Children (4)	Schizophrenia
Krieger, T.; Feron, F.; Dorant, E. (2020) Germany [29] #8	Two-level multi-methodological evaluation of a new complex primary support program for stroke caregivers in Germany	*n* = 62	Stroke

**Table 2:** Methodological approach and results of the studies selected for analysis.

Reference	Methodological Approach	Results	Study Quality
[22] #1	Quantitative Comparative study Questionnaire Revised Scale for Care-giving Self-Efficacy; 14-item Psychological Distress Index; The Caregiver Reaction Assessment; SF-36 Descriptive and inferential statistics	Caregiver’s confidence: request respite care: 51.14 (EOD ^1^), 45.93 (LOD ^2^); cope with behaviors: 73.96 (EOD), 71.15 (LOD); control disturbing thoughts: 65.41 (EOD), 73.28 (LOD); role confidence: 51.47 (EOD), 50.30 (LOD) Caregiver’s level of distress: psychological distress: 26.17 (EOD), 23.90 (LOD); anxiety: 8.79 (EOD), 7.74 (LOD); depression: 5.89 (EOD), 5.64 (LOD); impact of caregiving experience: caregiver’s esteem: 27.70 (EOD), 26.98 (LOD)	Moderate
[23] #2	Pre- and post-test study (after implementation of Respite Educational and Support Tools REST) Modified version of the instrument developed by the ARCH National Respite Network and Resource Center (NRNRC) ^3^ ANOVA	Well-being indicators: stress levels: 3.31 ± 1.24 (BR ^4^), 2.36 ± 0.94 (DR ^5^); general health problems status: 2.56 ± 1.19 (BR), 1.9 ± 1.00 (DR); social/recreational activities: 2.02 ± 0.96 (BR), 2.88 ± 0.98 (DR); placing the care recipient in out-of-home care: 1.83 ± 1.12 (BR), 1.46 ± 0.82 (DR); stress-related health symptoms: 5.24 ± 3.70 (BR), 3.06 ± 3.09 (DR)	Moderate
[24] #3	Quantitative Chinese e-Health Literacy scale (C-eHEals); short version of Zarit Burden Interview Linear regression	Means of caregiver e-health literacy: 2.89 ± 1.23; means of caregiver burden: 2.14 ± 1.10; positive association between e-health literacy and caregiver burden (β = 0.14; 95% confidence interval: [0.03, 0.27]).	Moderate
[25] #4	Quantitative Caregiver Appraisal Scale TBI-Care Quality of Life (QoL) ^6^; Talking Touchscreen Technology (Health LiTT) ^7^; General Health Status (SF-12) Linear regression	High health literacy: 78.6%; low health literacy: 21.4%; low literacy group significant effects: subjective caregiving burden (*p* = 0.041), relationship satisfaction with the patient (*p* = 0.028), caregiving mastery (*p* = 0.030)	Moderate
[26] #5	Cross-sectional descriptive design City of Hope Quality of Life (Family Version) Descriptive statistics Linear regression	Mean score QoL: 180.24 ± 22.90 Lowest QoL scores: physical well-being (26.5 ± 7.9); spiritual well-being (35.5 ± 10.7)	High
[27] #6	Cross-sectional survey Stress Level score scale; Quality of Life level score scale. Correlational and descriptive statistics	Mean stress score = 3.56; mean score for each QoL variable > 3; significative stress and psychosocial overload	High
[28] #7	Qualitative Unstructured interview Thematic analysis	Emergent themes: lack of knowledge about schizophrenia; emotional overload; anxiety about the future	Low
[29] #8	Multi-methodological in 2 levels, with 2 connected simultaneous studies and a sequential exploratory draw Pre- and post-test Questionnaire and interview Freebody and Luke’s Health Literacy framework Psycho-social health: 6 items to measure sense of certainty and 4 items for life balance	Health literacy (HL): functional HL (knowledge) [3.7 ± 0.8 (T0), 4.2 ± 0.8 (T1)]; interactive HL (capability to act) [3.3 ± 1.0 (T0), 3.7 ± 0.8 (T1)]; critical HL (individual empowerment) [3.0 ± 0.9 (T0), 3.2 ± 0.8 (T1)] Psycho-social health: sense of certainty [3.0 ± 0.8 (T0), 3.2 ± 0.7 (T1)]; life balance [2.8 ± 0.9 (T0), 2.9 ± 0.9 (T1)]	Moderate

^1^ Early-onset dementia. ^2^ Late-onset dementia. ^3^ Evaluates caregivers well-being. ^4^ Before respite. ^5^ During respite. ^6^ Caregiver health-related quality of life. ^7^ Health Literacy Assessment.

## Data Availability

Not applicable.
